# Reducing inequalities in Mental Health Services - how to be more culturally sensitive in a culturally diverse world?

**DOI:** 10.1192/j.eurpsy.2025.1281

**Published:** 2025-08-26

**Authors:** M. Andrade, F. Ramalheira, M. Magalhães

**Affiliations:** 1 Hospital Júlio de Matos - Unidade Local de Saúde São José, Lisbon, Portugal

## Abstract

**Introduction:**

As psychiatrists, we provide care to patients from diverse cultural backgrounds, whose mental health is often affected by migratory trajectories and post-migration factors in the destination country, such as exposure to adversities and inequalities in access to healthcare. Moreover, culture plays a significant role in the presentation of symptoms and influences explanatory models of illness, health objectives, resources mobilized, and treatment styles. Thus, the social determinants of health are often distinct from those of the community of origin.

**Objectives:**

The development of culturally sensitive and adapted services becomes essential to reduce healthcare inequalities. We intend to review the concept of cultural diversity and explore various models studied for intervening in mental health care in culturally diverse communities.

**Methods:**

Narrative literature review through bibliographic research and publications in the Pubmed and Google Scholar databases, using the terms “cultural psychiatry,” “cultural competence,” and “migration.”

**Results:**

Some barriers to healthcare access for the migrant population include ethnolinguistic barriers, stigma, resilience and minimization of symptoms and complaints, and issues related to accessibility and service costs. On the other hand, healthcare professionals also identify bureaucratic issues and report a lack of knowledge regarding different cultural practices, showing interest in obtaining more training in the area. Some strategies to adapt mental health services to cultural diversity include health literacy, “ethnic matching” techniques, interpreter and cultural mediator training, and building cultural competence for both healthcare professionals, institutions and healthcare systems. Cultural formulation uses specific tools to gather clinical information and cultural context, showing positive preliminary results.

**Conclusions:**

Cultural competence represents a comprehensive response to the needs of migrant and culturally diverse population, requiring the acquisition of knowledge and skills at both the individual and organizational levels. Investing in training and research on cultural competence will further enhance the effectiveness and accessibility of mental health care, contributing to the reduction of inequalities and better health outcomes.

**Disclosure of Interest:**

None Declared

